# Network Pharmacology Deciphering Mechanisms of Volatiles of* Wendan* Granule for the Treatment of Alzheimer's Disease

**DOI:** 10.1155/2019/7826769

**Published:** 2019-02-12

**Authors:** Jun-feng Liu, An-na Hu, Jun-feng Zan, Ping Wang, Qiu-yun You, Ai-hua Tan

**Affiliations:** ^1^Ministry of Education Key Laboratory of Chinese Medicine Resource and Compound Prescription, Hubei University of Chinese Medicine, Wuhan 430065, China; ^2^Pharmacy School, Hubei University of Chinese Medicine, Wuhan 430065, China; ^3^Institute of Geriatrics, Hubei University of Chinese Medicine, Wuhan 430065, China

## Abstract

**Objective:**

To explore the mechanisms of the volatiles of* Wendan* granule (WDG) for the treatment of Alzheimer's disease, network pharmacology method integrating absorption, distribution, metabolism, and excretion (ADME) screening, target fishing, network constructing, pathway analysing, and correlated diseases prediction was applied.

**Methods:**

Twelve small molecular compounds of WDG were selected as the objects from 74 volatiles with the relative abundances above 2 %, and their ADME parameters were collected from Traditional Chinese Medicine Systems Pharmacology platform (TCMSP), and the corresponding targets, genes, pathways, and diseases were predicted according to the data provided by TCMSP, DrugBank, Uniport, and the Database for Annotation, Visualization, and Integrated Discovery (DAVID). Then the related pathways and correlation analysis were explored by the Kyoto Encyclopedia and Genomes (KEGG) database. Finally, the networks of compound target, target pathway, and pathway disease of WDG were constructed by Cytoscape software.

**Results:**

Twelve compounds interacted with 49 targets, of which top three targets were gamma-aminobutyric acid receptor subunit alpha-1 (GABRA1), prostaglandin G/H synthase 2 (PGHS-2), and sodium-dependent noradrenaline transporter. Interestingly, these targets were highly associated with depression, insomnia, and Alzheimer's disease that mainly corresponded to mental and emotional illnesses.

**Conclusion:**

The integrated network pharmacology method provides precise probe to illuminate the molecular mechanisms of the main volatiles of WDG for relieving senile dementia related syndromes, which will also facilitate the application of traditional Chinese medicine as an alternative or supplementary to conventional treatments of AD, as well as follow-up studies such as upgrading the quality standard of clinically applied herbal medicine and novel drug development.

## 1. Introduction

Alzheimer's disease (AD), also known as senile dementia, is an age-related progressive neurodegenerative disease that continues to form a huge challenge to the aging community, especially a heavy burden for patients and their family. With the worldwide reduction in birth rates and prolonged life span expectancies, the Alzheimer's disease together with other dementias was considered to be one of the 10 leading causes of disability among people with the age above 60 globally [1]. The decline of cognitive function of old people happened with the progress of aging; thus, the early detection and early intervention in cognitive dysfunction are important for delaying or preventing the occurrence or progression of dementia, enabling patients to maintain basic cognitive functions and improve their quality of life for a longer period of time [2]. Various medicines have been developed all over the world for the treatment of AD. There are four conventional therapeutics strategies for the treatment of AD using modern clinical medicines: (1) restoration of cognitive impairment, (2) activation of *α*-secretase, (3) inhibition of *β*-secretase and *γ*-secretase, and (4) inhibition of Tau hyperphosphorylation [3]. However, for clinically applied medicines, such as (1) acetylcholinesterase inhibitors: Donepezil, Galantamine, (2) N-methyl-D-aspartate acid receptor: Magnesium Hydrochloride [4], and (3) ABT-126 [5] that only target the symptoms of AD, but not the pathogenesis. Though a certain degree of recovering impairment efficacies these medicines may have, their side effects may also have to be considered. Gastrointestinal reactions, diarrhoea, nausea and vomiting, insomnia, fatigue, and urinary incontinence are the common adverse effects of cholinergic drugs. Magnesium Hydrochloride, which is the only nonacetylcholinesterase inhibitor approved for AD treatment, has the adverse effects such as fatigue, high blood pressure, dizziness, headache, and constipation.

Traditional Chinese medicine (TCM) prescriptions play an important role in the treatment of various serious diseases, especially those the western medicine find it difficult to tackle, because the therapeutic effects of TCM are based on synergistic and holistic theory. Unfortunately, the uncertainty of potential active compounds, explicit targets, and underlying pharmacological mechanisms impeded the modernization of TCM. Network pharmacology [6], an emerging promising subject and an advanced approach to new drug discovery, provides novel tactics for the understanding of the relationship between drugs and diseases at a systematic level.

*Wendan* granule (WDG) is a hospital preparation of traditional Chinese medicine prescription for the treatment of AD. It is a modern dosage form produced based on a modified prescription of* Wendan* decoction, which is an ancient and classical prescription with the function of “*Hua-Tan* (化痰)”. TCM believes that* Tan* can stay in various parts of the body, including brain, and produce all sorts of diseases. On the other hand, just like the aggregated *β*-amyloid in brains of AD patients,* Tan* itself is also a pathological product of various diseases. Therefore, the pharmacological efficacies of WDG are predicted to be reducing the production and promoting the clearance of *β*-amyloid in brains of AD patients by multiple mechanisms. Our previous study has identified 74 volatiles by employing HS-SPME-GC-MS [7]. However, their specific mechanisms of efficacy are still vague. Hence, in this study, network pharmacology was applied to explore the mechanisms of the main volatiles of WDG for the treatment of AD.

## 2. Materials and Methods

### 2.1. Materials

#### 2.1.1. Analysis Platforms and Databases

TCMSP (http://lsp.nwu.edu.cn/index.php) is a systems pharmacology platform and database of Chinese herbal medicines [8]. DrugBank (https://www.drugbank.ca/) is a bioinformatics and cheminformatics resource database [9]. Uniprot (https://www.uniprot.org/) is a comprehensive database about protein [10]. DAVID (https://david.ncifcrf.gov/) is a database to help understand biological meaning behind genes by providing various functional annotations [11]. KEGG (https://www.kegg.jp/) is Kyoto Encyclopedia of Genes and Genomes [12]. GeneMANIA (http://genemania.org/) is a website used for predicting the function of genes and gene sets [13].

#### 2.1.2. Utility Software

ChemBioOffice Ultra 12.0 (Perkin Elmer Inc., Waltham, MA, USA) is applied for candidate compounds structures constructing. Cytoscape 3.5.1 is open source software for visualizing complex networks and integrating them with all sorts of types of attribute data [14].

### 2.2. Methods

#### 2.2.1. Candidate Components Screen

Our previous study has identified 74 volatiles from WDG by employing HS-SPME-GC-MS. In order to acquire the potential main volatile compounds from the granule, four screening criteria were defined as follows: OB (oral bioavailability)* ⩾*30%, Caco-2 permeability>0, BBB (blood–brain barrier [15])* ⩾*0.3, and relative abundance (RA)* ⩾*0.2. Based on the ADME parameters in TCMSP and RA values obtained from previous work [7], the volatiles which satisfied the principles were selected as the candidate compounds.

#### 2.2.2. Targets Screening

To identify the corresponding targets of main compounds of WDG, several approaches were implemented. First of all, TCMSP and DrugBank database were applied to find out the potential targets. Then, “drug-target” network will be constructed by Cytoscape 3.5.1 software. The candidate targets were mainly screened by degree that represents the number of edges adjacent to a node [3]. Next, for more accurate result obtaining purpose, the targets with degree* ⩾*3 were chosen as candidate targets and others were eliminated.

#### 2.2.3. GeneMANIA Analysis

GeneMANIA was used for predicting the function of genes and gene sets. A relationship network of genes was given after input of a set of gene list and species selected as “homo sapiens.”

#### 2.2.4. GO and Pathway Annotation

The targets were input to the DAVID for further investigation such as Gene Ontology (GO), pathways. Construct the “target-pathway” network. The pathways that are equal to or above degree 3 were analysed for candidate pathways identification.

#### 2.2.5. Identification for Diseases

The KEGG gave you information about related disease when candidate pathways entered and then constructed the “pathway-disease” network and preliminarily speculate the pharmacological mechanisms of WDG with all the information above.

## 3. Results and Discussion

### 3.1. Identification of Active Volatility Components

**Figure 1:**
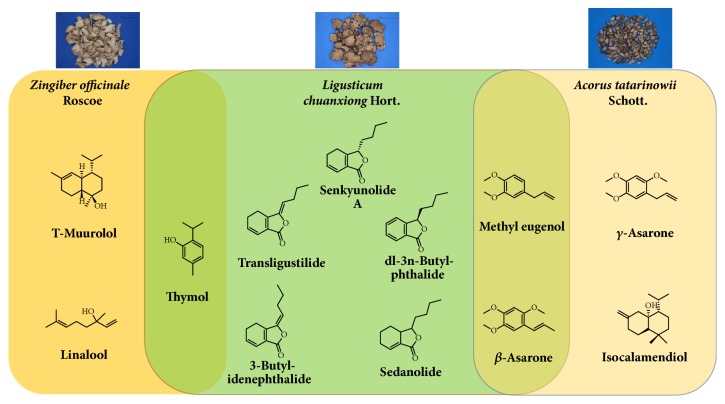
Twelve candidate compounds and their corresponding herbal medicines.

From the former work, 75 volatility components were identified by employing HS-SPME-GC-MS. For the purpose of acquiring the main compounds, four screening criteria were defined as follows: OB*⩾*30%, Caco-2 permeability>0, BBB*⩾*0.3, and RA*⩾*0.2. A total of 12 compounds including *γ*-Asarone, trans-ligustilide, and senkyunolide A, which showed poor OB but have high abundance, were selected as the candidate compounds for more accurate investigation (Table 1, see Table 1S in the Supplementary Material for the detailed information of the twelve candidate volatiles). For the purpose of tracing back to the original herbal medicines applied to form the WDG prescription, twelve compounds were input into TCMSP to backtrack the corresponding herbs (Figure 1), and two, five, and two were recognized as those of* Ginger*,* Chuanxiong*, and* Acorus tatarinowii*, respectively. Senkyunolide A, one of the main bioactive constituents in the herb* Rhizoma Chuanxiong*, shows protective effect on the injury of central nervous system and on contractions to various contractile agents in rat isolated aorta [16]. *β*-Asarone, which shows the highest abundance in WDG and a shared compound of* Chuanxiong* and* Acorus tatarinowii*, has been investigated with regard to effects on central nervous system [15, 17]. Thymol, a common ingredient shared by* Ginger* and* Chuanxiong*, possesses active antioxidant effect [18]. Butylidenephthalide has been suggested to produce various pharmacological activities in cerebral blood vessels [19]. Butylphthalide could decrease the brain infarct volume and enhance microcirculation, thus benefiting patients [20]. BBB of all the 12 are more than 1.00 which means they all have effect to break through the BBB to cure disease related to CNS.

### 3.2. Analysis of “Compound-Target” Network

**Figure 2:**
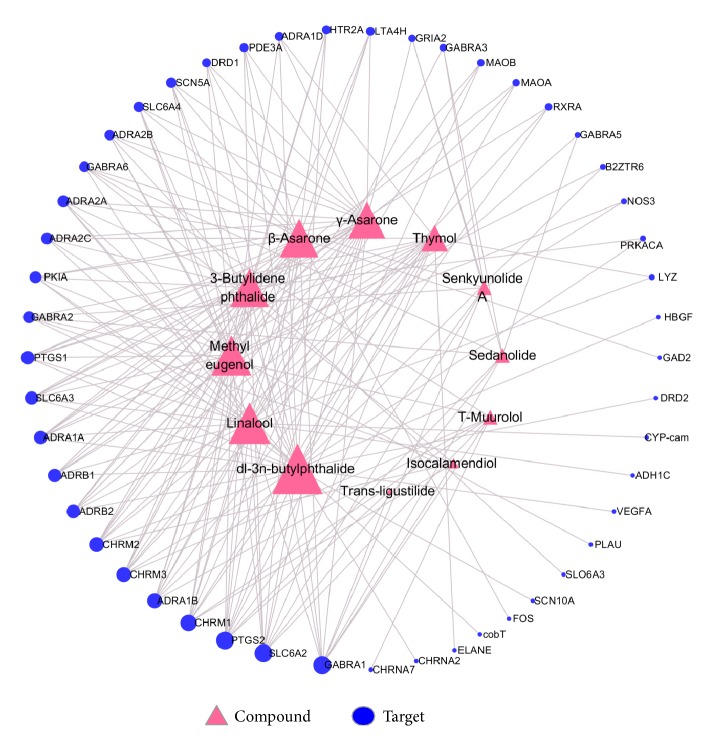
Compound-target network analyses results. A compound and a target linked if the target protein was hit by the corresponding compound. Node size was proportional to its degree that correlated with the number edges of each target/compound. 12 candidate compounds were linked with 49 candidate targets. The network shows that most of the compounds hit more than one target, and vice versa. (The detailed compound-target information of the twelve volatiles with their corresponding targets can be found in Table 2S_1-9 in the Supplementary Material.)

AD has been considered as one of the most serious threats of health, although many different types of therapeutic methods have been applied for the management and prevention of AD. Identifying the compound interacting with targets is a good strategy for drug discovery. TCMSP and DrugBank database were applied for predicting the potential targets for each compound. As a result, 201 compound-target interactions were identified between 12 compounds and 49 targets, and the candidate targets were selected according to the degrees that reflect the number of edges of each target (Figure 2, see Table 2S_1-9 in the Supplementary Material for the detailed compound-target information of the twelve candidate volatiles with their corresponding targets). After calculating the value of degree for each target in C-T network, the average value of the degrees was 4.10. Therefore, the targets with degree*⩾*3 were regarded as candidate targets for further investigation (Table 1).

GABRA1 shows the highest degree, followed by PTGS2, SLC6A2, and CHRM1. Many of them have been verified in previous researches. For instance, GABRA1 was found to play an important role in Alzheimer's dementia [21], and a previous study reported that gamma-amino butyric acid receptor subunit alpha-1 could inhibit neurotransmission in the brain [22]. GABA receptors might induce a significant impact on brain structures and functions and might also participate in the dysregulation of the balance between excitatory and inhibitory neurotransmission that was observed in AD patients [23]. Both PTGS1 and PTGS2 could convert arachidonate into prostaglandin H2 (PGH2). PTGS1, which is involved in the production of prostanoids particularly in the stomach and platelets, could help promote platelet activation and aggregation, vasoconstriction, and proliferation of vascular smooth muscle cells. PTGS2 was mainly expressed in endothelium, kidney, and brain and in cancers. Some researchers reported that patients with AD have a higher PTGS2 in hippocampal neurons, and it is convinced that PTGS2 played an important role in the pathogenesis of AD for it could convert arachidonate into PGH2 which might induce inflammation in the brain [24–26]. Meanwhile, the modern epidemiology also showed that the use of nonsteroidal anti-inflammatory drugs can reduce the incidence of AD; therefore, controlling the inflammatory response became one of the important therapeutic strategies of clinical treatment of AD [27]. SLC6A2 was closely associated with depression. This protein was the target of psychomotor stimulants such as Amphetamines or Cocaine [28, 29]. CHRM1, CHRM2, and CHRM3 were three subtypes of CHRM (muscarinic acetylcholine receptors), one of cholinergic neurotransmitter receptors, that usually participated in the regulation of cognitive function [30, 31]. Relevant pharmacological experiments have indicated the specific reduction of cholinergic neurons on basal forebrain in AD patients, and the rate of hippocampal atrophy in AD patients was halved after cholinesterase inhibitors were given for one year [32]. CHRM1 was considered as a key target of AD therapy. The function of CHRM2 was to control the release of acetylcholine which is located in the terminal cholinergic neurons in the forebrain [33]. CHRM3 was able to promote various cellular activities by regulating the passage of different signals [34]. In summary, the common functions of these targets were relevant with nervous system diseases like insomnia, AD, which were also the indications of WDG. The relationship between the compounds and targets revealed the truth that multiple compounds and multiple targets interact with each other in molecular system that might break off and jump out of the traditional “one-target-one-compound” model.

### 3.3. Gene Function Analysis by Using GeneMANIA

**Figure 3:**
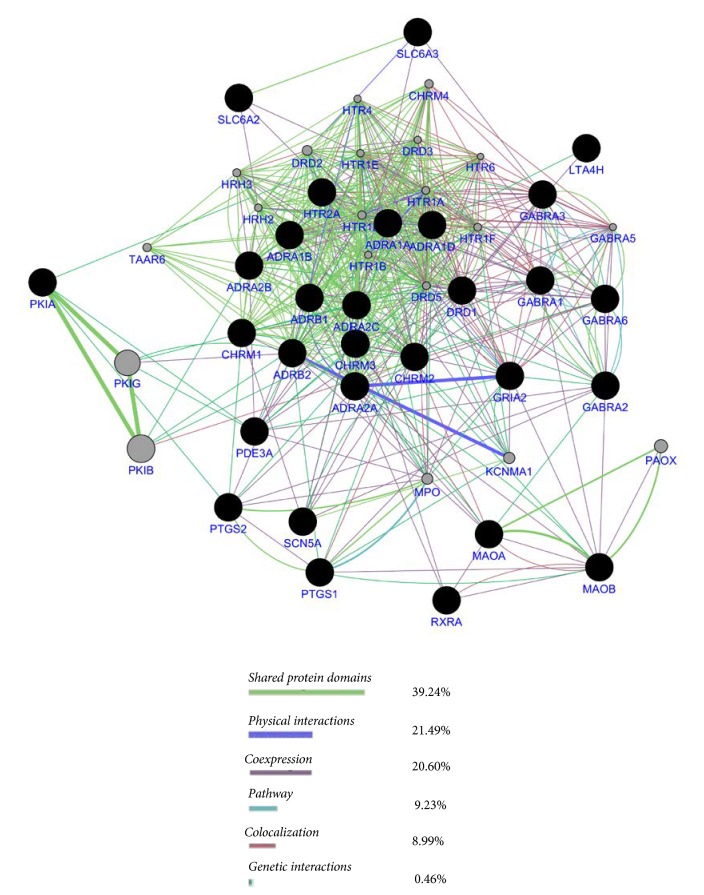
GENEMAIA based network analysis: the black nodes represented the input genes and the grey nodes represented the associated genes. The edges with different colour were associated with different functions. (The detailed information of the integrated GO enrichment analysis results of thirty selected candidate targets can be found in Table 3S_1-3 in the Supplementary Material.)

Most successful computational methods for compound interacting with targets prediction integrate the prediction of multiple direct targets and multiple indirect targets. GeneMANIA, a useful website to find genes most related to the query genes, is capable of predicting protein functions with the advanced and unique algorithm and is also regarded as a real-time multiple association network integration algorithm for predicting gene function such as coexpression, colocalization, pathways, and protein domain [35].  Figure 3 shows the network generated by GeneMANIA website. The nodes with black colour represent the input genes and the grey nodes represent the associated genes. The edges with different colour are associated with different functions. As shown in the results (Figure 3), 39.24% of the genes shared protein domains and 20.60% had the coexpression.

### 3.4. Analysis of GO Enrichment

Thirty candidate targets were chosen for further investigation by using DAVID (Table 3, see Table 3S_1-3 in the Supplementary Material for the integrated GO enrichment analysis results of thirty selected candidate targets). Gene ontology enrichment analysis consisted of three parts, BP (biological process), CC (cellular component), and MF (molecular function). Drug binding, protein heterodimerization activity, and epinephrine binding were predicted to be the main biological functions induced by 12 volatiles. Plasma membrane, integral component of plasma membrane, and integral component of membrane were ranked as top three cellular components, which might reflect that most of the small volatiles were targeted to neural cells. The top three biological processes were the G protein coupled receptor signaling pathway, the acetylate cycles activating adrenergic receptor signaling pathway, and the response to drug. G protein coupled receptor signaling pathway was ranked as No. 1, which indicated that G protein coupled receptor might be one of the main drug targets for the treatment. There were previous studies which reported that G protein coupled receptors might serve as the largest pharmacodynamic therapeutic target for AD, because they can directly affect the beta-amyloid signaling cascade in the nervous system by regulating *α*-, *β*-, *γ*-, secretory enzyme secretion, amyloid precursor protein (APP) production, and A*β* degradation[36]. Furthermore, CHRM1, CHRM3, and HTR2A all belonged to the G protein coupled receptor. The abnormality of a variety of signal pathways and signal transmission played important roles in the pathogenesis of AD; moreover, the dysfunction of adenylate cyclase signaling system was considered to be the main cause of AD. G protein-mediated dysfunction of adenylate cyclase signaling system was an important enlightenment for the prevention and treatment of AD [37]. Interestingly, ADRA1B and ADRA1A, an alpha-adrenergic receptor, mediated their effects through binding to the G protein that could activate the phosphatidylinositol-calcium second messenger system [38]. ADRB1 and ADRB2 were *β*-adrenergic receptors that mediate catecholamine-induced activation of adenylate cyclase through the sensitization of the G protein [39]. ADRA2A and ADRA2C were alpha-2 adrenergic receptors that mediated catecholamine-induced inhibition of adenylate cyclase also through the sensitization of the G protein [40].

### 3.5. Target-Pathway and Pathway-Disease Networks

**Figure 4:**
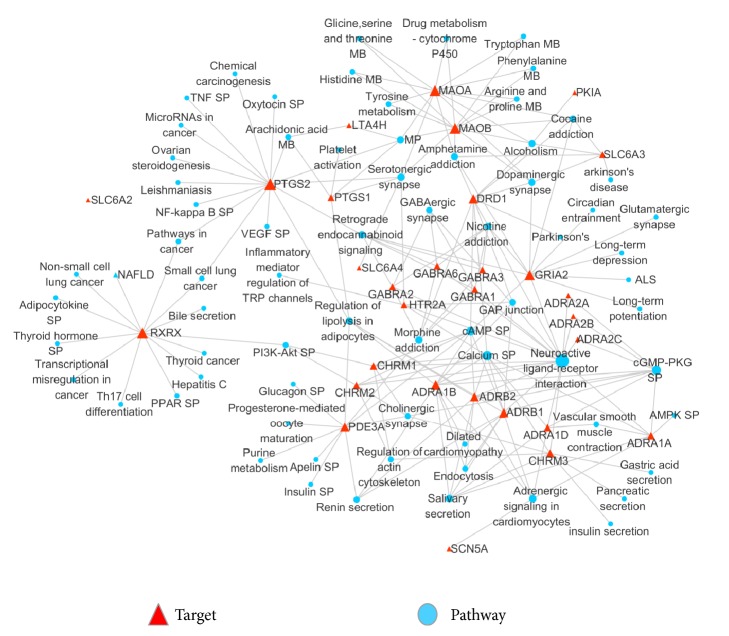
Target-pathway network analyses results. The red triangle nodes represented the targets while the blue circle nodes represented the pathways. The target proteins were linked with their corresponding pathways. Nodes sizes were proportional to their degrees (abbreviations: SP: signaling pathway; MB: metabolism; NAFLD: nonalcoholic fatty liver disease; and ALS: amyotrophic lateral sclerosis.).

For the purpose of systematically deciphering the multiple underlying mechanisms of volatiles of WDG, all of the pathways interacting with candidate targets were extracted from KEGG pathways database using DAVID, and then the “target-pathway” network was constructed (Figure 4). Twenty-three related pathways were found, including neuroactive ligand-receptor interaction, calcium signaling pathway, cGMP-PKG signaling pathway, and cAMP signaling pathway. Signaling pathways have been regarded as one of the most important parts of systems pharmacology [41]. As shown in Figure 3, five targets including DAD1, GRIA2, MAOA, MAOB, and SLC6A3 were all found to be associated with Cocaine addiction, Amphetamine addiction, Dopaminergic synapse, and Alcoholism together, which could help speculate the pharmacokinetic synergistic effects among them. GABA family including GABRA1, GABRA2, GABRA3, and GABRA6 worked together on neuroactive ligand-receptor interaction, Morphine addiction, retrograde endocannabinoid signaling, Nicotine addiction, and GABAergic synapse pathways. ADRA1A, ADRA1B, ADRA1D, ADRB1, and ADRB2 that belonged to adrenergic receptor were all associated with neuroactive ligand-receptor interaction, calcium signaling pathway, cGMP-PKG signaling pathway, salivary secretion, and adrenergic signaling in cardiomyocytes. Neuroactive ligand-receptor interaction might be the main pathway that correlated with the mechanism. For all the pathways, three of them belonged to the cellular processes: regulation of actin cytoskeleton, gap junction, and endocytosis. Five belonged to environmental information processing or signal transduction: calcium signaling pathway, cGMP-PKG signaling pathway, cAMP signaling pathway, PI3K-Akt signaling pathway, and neuroactive ligand-receptor interaction. Five belonged to human diseases or substance dependence: Morphine addiction, Amphetamine addiction, Alcoholism, Cocaine addiction, and Nicotine addiction. Nine belonged to organismal systems: adrenergic signaling in cardiomyocytes, salivary secretion, regulation of lipolysis in adipocytes, renin secretion, retrograde endocannabinoid signaling, serotonergic synapse, dopaminergic synapse, GABAergic synapse, and cholinergic synapse while, the last four also belonged to nervous system.

**Figure 5:**
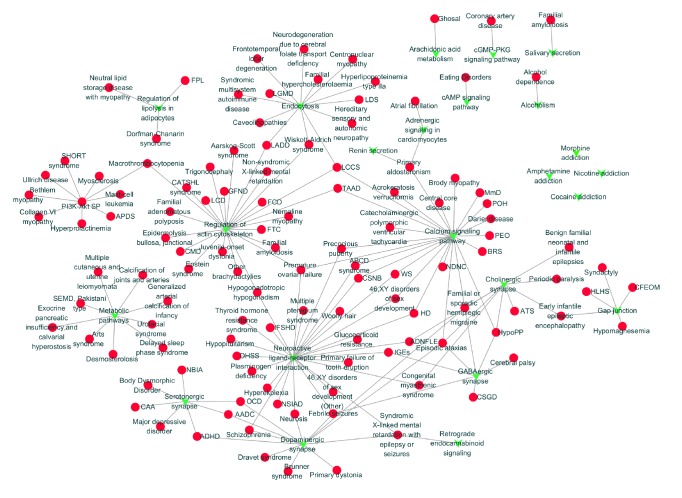
Pathway-disease network analyses results. Red nodes represented diseases while the green nodes represented pathways. The edges represented the interaction between them (abbreviations: AADC: aromatic L-amino acid decarboxylase deficiency; ADHD: attention deficit hyperactivity disorder; ADNFLE: autosomal dominant nocturnal frontal lobe epilepsy; APDS: activated PI3K-delta syndrome; ATS: Andersen-Tawil syndrome; BRS: Brugada syndrome; CAA: cerebral amyloid angiopathy; CFEOM: congenital fibrosis of the extraocular muscles; CMD/MDC: congenital muscular dystrophies; CSGD: congenital systemic glutamine deficiency; CSNB: congenital stationary night blindness; FCD: fleck corneal dystrophy; FPL: familial partial lipodystrophy; FTC: familial tumoral calcinosis; GFND: Glomerulopathy with fibronectin deposits; HD: Hirschsprung disease; HLHS: hypoplastic left heart syndrome; HypoPP: hypokalemic periodic paralysis; IFSHD: isolated follicle-stimulating hormone deficiency; IGEs: idiopathic generalized epilepsies; LADD: lacrimo-auriculo-dento-digital syndrome; LCCS: lethal congenital contracture syndrome; LCD: lattice corneal dystrophies; LDS: Loeys-Dietz syndrome; LGMD: limb-girdle muscular dystrophy; MmD: multiminicore disease; NBIA: neurodegeneration with brain iron accumulation; NDNC: nonsyndromic congenital nail disorder; NSIAD: nephrogenic syndrome of inappropriate antidiuresis; OCD: obsessive compulsive disorder; OHSS: ovarian hyperstimulation syndrome; PEO: progressive external ophthalmoplegia; POH: progressive osseous heteroplasia; TAAD: familial thoracic aortic aneurysm and dissection; and WS: Waardenburg syndrome).

In Figure 5, there were totally 142 nodes while 24 of which shaped green ‘V's corresponded to candidate pathways and the remaining 118 red circle nodes represented diseases. Neuroactive ligand-receptor interaction pathways, calcium signaling pathway, retrograde endocannabinoid signaling pathways, and endocytosis were corresponding to 27, 23, 23, and 13 kinds of different diseases. All the corresponding diseases were classified by KEGG while nervous system related disease accounts for largest proportion followed by musculoskeletal disease, developmental disorder, endocrine disease, inherited metabolic disease, and cardiovascular disease.

## 4. Discussion

TCM prescriptions were prescribed for fighting against diseases from ancient China till now based on the theories of traditional Chinese medicine. Multicomponents, multitargets, and various pharmacological actions within herbs were the superiority, but also the key issues of the modernization studies of traditional Chinese medicine. Here, we presented efficient tactics that contribute to understanding the pharmacological mechanism of TCM prescription by “network pharmacology” in molecular level to reveal the interaction between small molecule compounds with protein targets, pathways, and diseases. The “network pharmacology” was first proposed by Andrew L. Hopkins [42]. Different from the conventional drug discovery mode, the network pharmacology breaks through the “one-drug-one-target” mode to the synergy of multiple targets to identify the therapeutic mechanisms of TCM based on holistic research strategy of multicomponents, multitargets, and multidiseases [43].

Twelve main compounds with favourable ADME were selected from seventy-four volatiles of WDG identified using HS-SPME-GC-MS. Then online databases, TCMSP and DrugBank, were applied to obtain potential interaction targets with the pathways, followed by GO enrichment clustering analysis. The results showed some compounds and targets identified in this research that might be conductive to the therapy and prevention of Alzheimer's disease had already been reported previously, which highlighted the credibility of the ADME evaluation system and target predicting databases. One target was hit by multiple ingredients, which coincided with the holistic view and synergistic action theory of TCM. Prediction of potential targets might be the first step in drug discovery within the wide application of computational target fishing technology. As shown in Table 2, some targets had already been reported previously. For example, GABRA1 is the most important inhibitory receptor in brain that can produce inhibitory postsynaptic sites to inhibit neuronal discharge by acting on the chloride channel after combining with GABA. GABRA1 and GABRA2 are the components of the heteropentameric receptor for GABA, receptor of GABA (gamma-aminobutyric acid) that is an important inhibitory neurotransmitter in vertebrate brain and plays an essential role in the treatment of insomnia by inhibiting brain neurotransmitters excitement [44]. Moreover, molecular mechanisms of Alzheimer's disease treatment were made manifest based on the “compound-target” network, “target-pathway” network, and “pathway-disease” network of WDG.

## 5. Conclusions

Previous studies had identified that WDG can improve the learning and memory ability by repairing the damage of cholinergic system, inhibiting the cytokine levels of transition-activated microglia and reducing the level of transient phosphorylation Tau protein [45–47].

Based on the series of results from the database, platform, and software given, we could preliminarily predict that the mechanism of WDG involved a large network which contained drugs, targets, pathways, and diseases. The main targets were GABRA1, PTGS2, SLC6A2, CHRM1, ADRA1B, CHRM2, CHRM3, ADRA1A, ADRB1, ADRB2, PTGS1, and SLC6A3 that conducted with neuroactive ligand-receptor interaction pathways, signaling pathways, and metabolism pathways related to analgesic, anticonvulsant, and repair reduction of cholinergic neurons and eliminate the inflammatory response to reduce the secretion of neurotoxic inflammatory cytokine to cure diseases related to AD and insomnia.

This study chose a novel strategy for the systematic understanding of the molecular mechanisms of the main volatiles of WDG relieving senile dementia related syndromes, which could facilitate the application of TCM in modern medicine and prescription/dosage form optimization [48]. Further studies, such as molecular biological experiments and clinical investigations, have to be carried out to verify these mechanisms. It might be a long-term and arduous task, but only in this way could we scientifically elaborate and evaluate the mechanisms of efficacy of the clinically approved TCM prescriptions and promote the worldwide application of Chinese medicine [49].

## Figures and Tables

**Table 1:** Candidate compounds ADME values and molecular information.

Compound Name	Molecular Formula	Molecular Weight	RA (%)	OB (%)	Caco-2	BBB
Senkyunolide A	C_12_H_16_O_2_	192	7.271	26.56	1.3	1.34
Trans-ligustilide	C_13_H_18_O	190	6.62	23.5	1.28	1.2
dl-3n-butylphthalide	C_12_H_14_O_2_	190	6.273	47.9	1.3	1.32
3-Butylidenephthalide	C_12_H_12_O_2_	188	4.138	42.44	1.32	1.27
Methyl eugenol	C_11_H_14_O_2_	178	2.987	73.36	1.47	1.41
T-Muurolol	C_15_H_26_O	222	3.129	30.41	1.36	1.44
Sedanolide	C_12_H_18_O_2_	194	3.522	62.46	1.24	1.4
*γ*-Asarone	C_12_H_16_O_3_	208	14.658	22.76	1.5	1.33
*β*-Asarone	C_12_H_16_O_3_	208	15.798	35.61	1.45	1.24
Linalool	C_10_H_18_O	154	2.74	38.29	1.29	1.33
Isocalamendiol	C_15_H_26_O_2_	238	2.518	57.63	0.94	0.74
Thymol	C_10_H_14_O	150	2.125	41.47	1.6	1.68

**Table 2:** Information of candidate targets, their corresponding gene symbols, and their degrees of correlation with compounds.

Target	Gene symbol	Degree
Gamma-aminobutyric acid receptor subunit alpha-1	GABRA1	10

Prostaglandin G/H synthase 2	PTGS2	10

Sodium-dependent noradrenaline transporter	SLC6A2	10

Muscarinic acetylcholine receptor M1	CHRM1	9

Alpha-1B adrenergic receptor	ADRA1B	8

Muscarinic acetylcholine receptor M2	CHRM2	8

Muscarinic acetylcholine receptor M3	CHRM3	8

Alpha-1A adrenergic receptor	ADRA1A	7

Beta-1 adrenergic receptor	ADRB1	7

Beta-2 adrenergic receptor	ADRB2	7

Prostaglandin G/H synthase 1	PTGS1	7

Sodium-dependent dopamine transporter	SLC6A3	7

Alpha-2A adrenergic receptor	ADRA2A	6

Alpha-2C adrenergic receptor	ADRA2c	6

cAMP-dependent protein kinase inhibitor alpha	PKIA	6

Gamma-aminobutyric-acid receptor alpha-2 subunit	GABRA2	6

Alpha-2B adrenergic receptor	ADRA2B	5

Gamma-aminobutyric-acid receptor subunit alpha-6	GABRA6	5

Sodium channel protein type 5 subunit alpha	SCN5A	5

Sodium-dependent serotonin transporter	SLC6A4	5

5-hydroxytryptamine 2A receptor	HTR2A	4

Alpha-1D adrenergic receptor	ADRA1D	4

CGMP-inhibited 3′,5′-cyclic phosphodiesterase A	PDE3A	4

Dopamine D1 receptor	DRD1	4

Leukotriene A-4 hydrolase	LTA4H	4

Amine oxidase [flavin-containing] A	MAOA	3

Amine oxidase [flavin-containing] B	MAOB	3

Gamma-aminobutyric-acid receptor alpha-3 subunit	GABRA3	3

Glutamate receptor 2	GRIA2	3

Retinoic acid receptor RXR-alpha	RXRA	3

**Table 3:** GO enrichment analyses using database DAVID.

Category	Term	Count	%	P-Value	Benjamini
BP	response to drug	9	0.2	3.20E-08	3.10E-06
	G-protein coupled receptor signalling pathway	9	0.2	1.00E-04	2.80E-03
	adenylate cyclase-activating adrenergic receptor signalling pathway	8	0.2	1.00E-15	3.80E-13
	cell-cell signalling	7	0.1	4.00E-06	2.50E-04
	positive regulation of vasoconstriction	6	0.1	2.10E-09	3.90E-07
	signal transduction	6	0.1	4.60E-02	3.10E-01

CC	plasma membrane	23	0.5	1.70E-09	5.90E-08
	integral component of plasma membrane	20	0.4	6.90E-15	4.70E-13
	integral component of membrane	16	0.3	6.60E-03	3.40E-02
	postsynaptic membrane	8	0.2	3.20E-08	7.30E-07
	cell junction	8	0.2	5.90E-06	8.20E-05

MF	drug binding	6	0.1	1.80E-07	1.10E-05
	protein heterodimerization activity	6	0.1	1.10E-03	1.40E-02
	protein homodimerization activity	6	0.1	7.50E-03	8.00E-02
	epinephrine binding	5	0.1	1.10E-10	1.30E-08
	GABA-A receptor activity	4	0.1	4.30E-06	1.80E-04
	extracellular ligand-gated ion channel activity	4	0.1	2.40E-05	4.90E-04

## Data Availability

The data used to support the findings of this study are available from the corresponding author upon request.

## Supplementary Materials

Supplementary Materials The detailed information of the 12 volatile compounds (S1), compound-target analysis data (S2), and Gene Ontology enrichment analysis results (S3) used to support the findings of this study is included within the supplementary information files.
