# Supplementary material for: Network Pharmacology Deciphering Mechanisms of Volatiles of Wendan Granule for the Treatment of Alzheimer's Disease
Source: Evid Based Complement Alternat Med. 2019 Feb 12;2019:7826769. doi: 10.1155/2019/7826769 (PMC6390244; doi:10.1155/2019/7826769)
Supplement: Supplementary Materials — The detailed information of the 12 volatile compounds (S1), compound-target analysis data (S2), and Gene Ontology enrichment analysis results (S3) used to support the findings of this study is included within the supplementary information files. [file 7826769.f1.zip › 7826769.f1/Graphical Abstract- the detailed flowchart of our network pharmacological study procedure..pptx]

## Slide 1
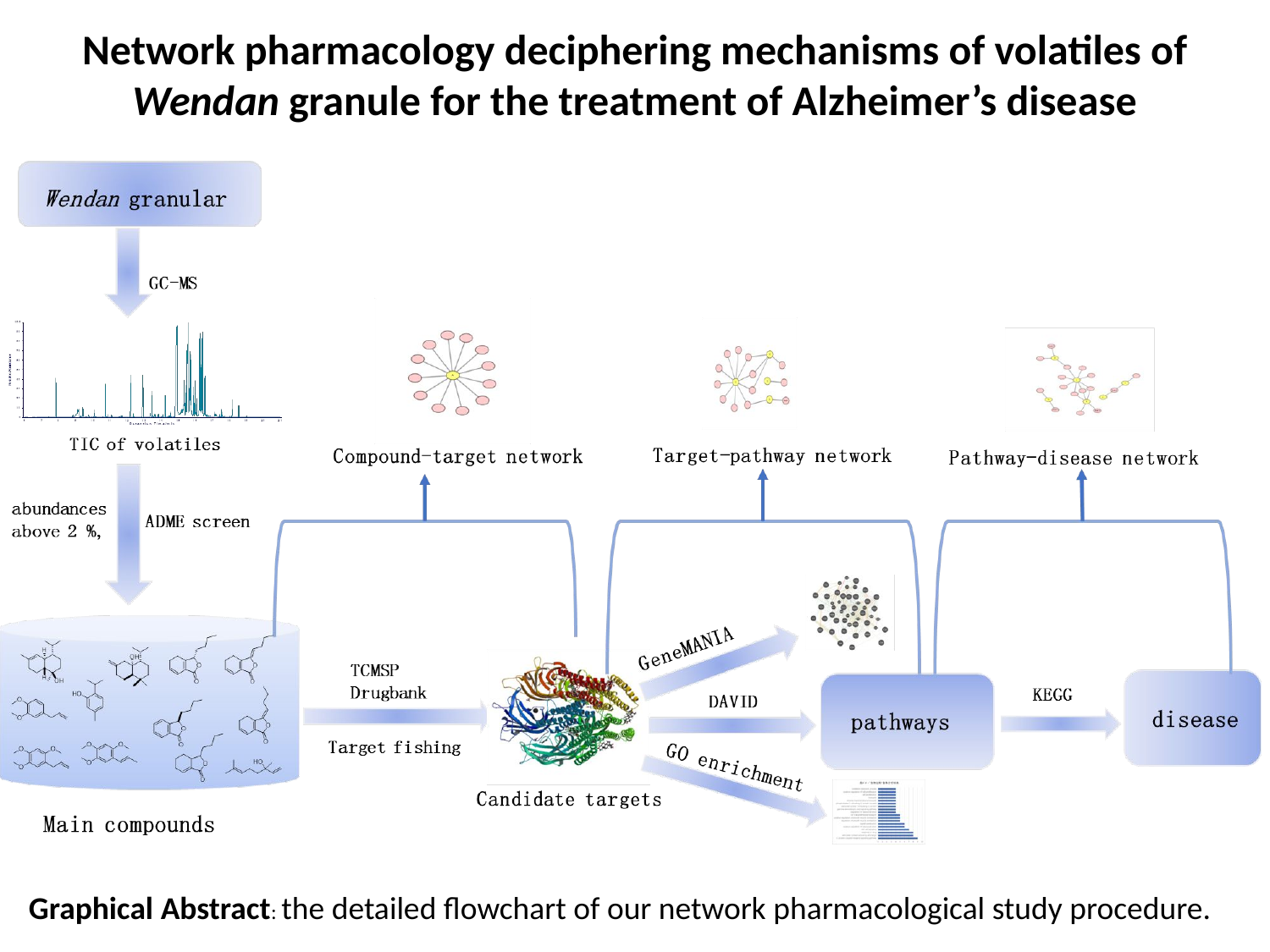

Network pharmacology deciphering mechanisms of volatiles of Wendan granule for the treatment of Alzheimer’s disease
Graphical Abstract: the detailed flowchart of our network pharmacological study procedure.
